# Supplementary figures and images for: Association of the PaO2/RDW ratio with 7-day mortality and risk of early invasive mechanical ventilation in ICU patients with delirium associated with ARDS: A retrospective cohort study from the MIMIC-IV database
Source: PLoS One. 2025 Dec 19;20(12):e0339390. doi: 10.1371/journal.pone.0339390 (PMC12716735; doi:10.1371/journal.pone.0339390)

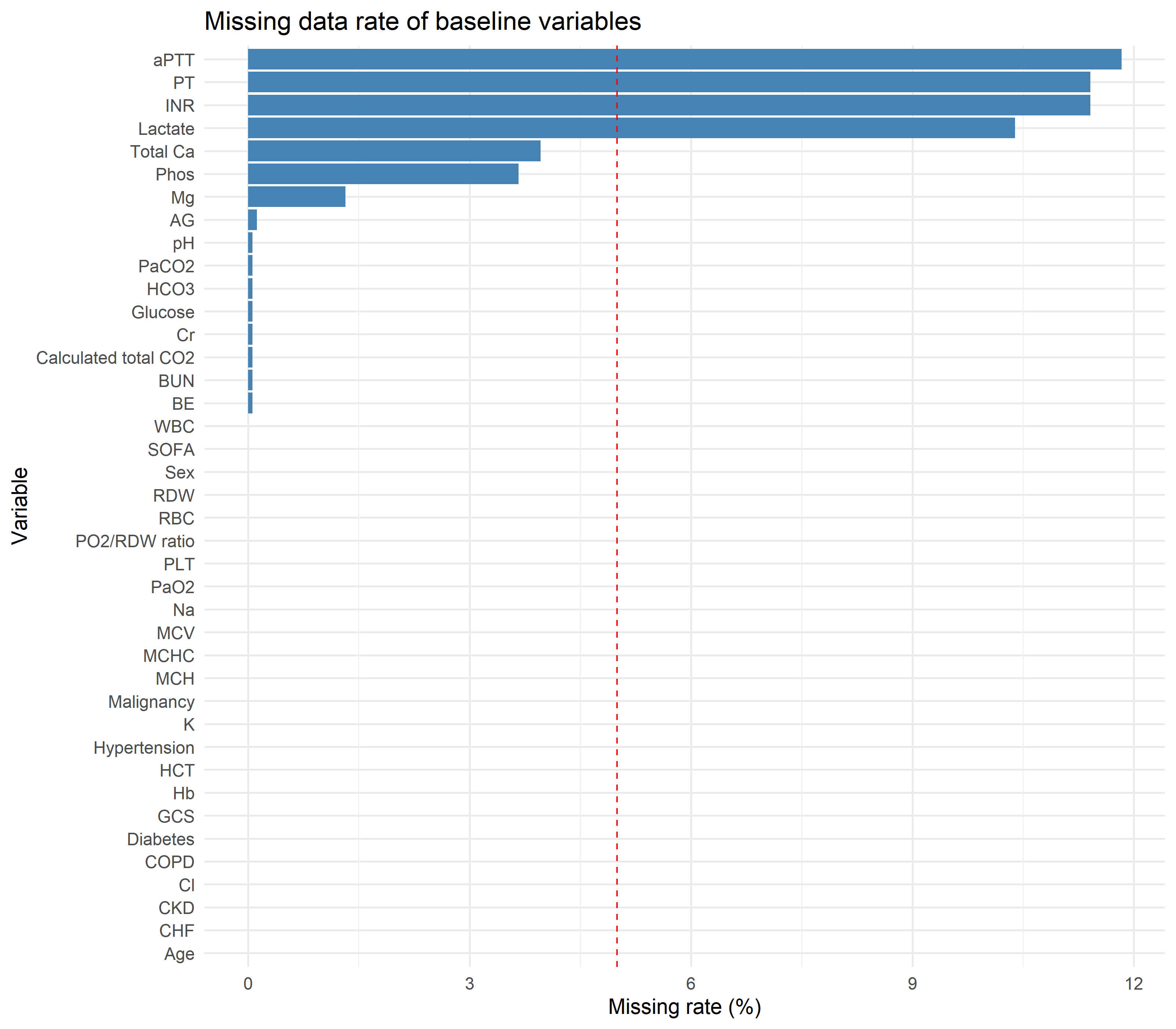

Supplement: S1 Fig — This figure illustrates the proportion of missing data for each clinical variable in the initial dataset. Variables with missing values exceeding 5% were excluded from the final analysis. (TIF) [file pone.0339390.s002.tif]
