# Supplementary material for: Association of the PaO2/RDW ratio with 7-day mortality and risk of early invasive mechanical ventilation in ICU patients with delirium associated with ARDS: A retrospective cohort study from the MIMIC-IV database
Source: PLoS One. 2025 Dec 19;20(12):e0339390. doi: 10.1371/journal.pone.0339390 (PMC12716735; doi:10.1371/journal.pone.0339390)
Supplement: Attachment — Submitted filename: Response to Reviewers.docx [file pone.0339390.s004.docx]

# Response to Reviewers

Manuscript ID: PONE-D-25-24350

Title: Association of the PaO₂/RDW Ratio With 7-Day Mortality and Risk of Early Invasive Mechanical Ventilation in ICU Patients With Delirium Associated With ARDS: A Retrospective Cohort Study From the MIMIC-IV Database

## General Response

We would like to express our sincere gratitude to the Academic Editor and the two reviewers for their valuable comments and suggestions. We have carefully revised the manuscript according to all feedback. The detailed responses to each comment are provided below, with corresponding revisions highlighted in the revised manuscript.

## Response to Reviewer #1

Comment: 1. In the abstract, line 3, it is better to use ‘’Poorly defined’’ instead of ‘’lacking’’.

Response: Revised accordingly. The word 'lacking' has been replaced with 'poorly defined'.

Comment: 2. In the abstract, line 5, I recommend using “delirium associated with ARDS in ICU patients” instead of “ARDS patients with ICU-acquired delirium.” The current phrasing may be misleading, as it suggests the delirium is primarily associated with ICU admission rather than with ARDS itself. Based on the manuscript, it appears that the authors intend to highlight delirium in the context of ARDS within the ICU setting. To avoid confusion and better reflect the study’s focus, the authors should revise this wording accordingly.

Response: Modified as suggested throughout the manuscript.

Comment: 3. While '’ICU'’ is widely recognized by clinicians, defining the term in the abstract is recommended to ensure clarity for all readers.

Response: Added the definition 'Intensive Care Unit (ICU)' at first mention.

Comment: 4. In the abstract, a definition of the MIMIC-IV database should be provided.

Response: Revised as suggested. We have added the full name and definition in the Abstract: “Data were extracted from the Medical Information Mart for Intensive Care IV (MIMIC-IV, version 3.1), a large, publicly available critical care database that contains de-identified health records of patients admitted to Beth Israel Deaconess Medical Center”.

Comment: 5. In the abstract, it is recommended to mention that a total of 4,116 patients were initially identified from the MIMIC-IV database, rather than only stating that the final cohort consisted of 1,508 patients. Including this information provides clearer context on the study population and the selection process.

Response: Added this information in the Abstract for clarity.

Comment: 6. In the abstract, conclusions, it's preferable to state that the PO2/RDW ratio may offer predictive insights, rather than claiming it is a reliable biomarker for predicting 7-day mortality and guiding mechanical ventilation in ARDS patients with delirium.

Response: We appreciate the reviewer’s suggestion. Since this study is observational and demonstrates associations rather than causal effects, we have revised the conclusion to adopt a more cautious tone. The relevant sentence now reads:

“The PaO₂/RDW ratio was independently associated with 7-day mortality after delirium onset and with the early risk of invasive mechanical ventilation among ICU patients with delirium associated with ARDS. As an easily obtainable composite index, the PaO₂/RDW ratio may serve as a convenient and informative biomarker for early risk assessment and clinical decision-making in critical care settings”. In addition, we have also revised the title and other relevant sections accordingly to maintain consistency with this adjustment.

Comment: 7–12. Improve Introduction, Methods, Results, Discussion, Conclusion.

Response: All sections have been substantially revised: Introduction expanded with more literature, Methods clarified (especially inclusion/exclusion), Results restructured with detailed figure legends, Discussion strengthened with additional references, and Conclusion rewritten for conciseness.

## Response to Reviewer #2

Comment: 1. Inconsistent, and potentially incorrect, interpretation of hazard ratios in the Abstract / Results.

Response: We appreciate the reviewer’s careful observation. We have rechecked all hazard ratios and confirmed that the reference group was set as the highest tertile of the PaO₂/RDW ratio, representing patients with relatively normal oxygenation and RDW status. Since lower PaO₂/RDW values reflect more severe hypoxemia and systemic stress, the lowest tertile corresponds to worse physiological conditions and higher mortality risk. Accordingly, all HRs and their interpretations have been corrected to reflect this directionality (i.e., lower PaO₂/RDW associated with higher mortality).

Comment: 2. Ambiguity about timing of exposure and outcome (reverse causation / temporality).

Response: We thank the reviewer for highlighting this important issue. To clarify the temporal relationship, we revised the SQL extraction process to include only PaO₂ measurements obtained within the first 24 hours of ICU admission and prior to the initiation of mechanical ventilation. Specifically, we extracted the exact timestamp of each PaO₂ measurement and used it to ensure that only values recorded before ventilation start time were retained. Consequently, the number of eligible patients changed slightly after this refinement, but the overall results and conclusions remained consistent.

Comment: 3. Confounding by indication and inappropriate adjustment for mediators.

Response: Ventilation was removed from mortality models to avoid collider bias.

Comment: 4. Proportional hazards (PH) assumption handling is inadequate.

Response: We thank the reviewer for this valuable comment. Instead of removing variables that violated the proportional hazards assumption, we incorporated time-dependent covariates using the tt() interaction function in the Cox model. After adding these interaction terms, the proportional hazards assumption was satisfied.

Comment: 5. Events-per-variable (EPV) and risk of overfitting.

Response: We appreciate the reviewer’s insightful comment. In accordance with your suggestion, we applied LASSO and Boruta feature selection methods to reduce variable redundancy (Figure 3A–D). After variable selection, the final Cox model achieved an events-per-variable (EPV) value of approximately 13.9, which meets the commonly accepted threshold for model stability and reliability.

Comment: 6. Missing data handling is unclear.

Response: We appreciate the reviewer’s comment. Variables with more than 5% missing values were excluded. For the remaining variables, we performed multiple imputation using the mice package in R. The imputation method was predictive mean matching (pmm) for continuous variables and logistic or polytomous regression for categorical variables. We used five iterations and generated one completed dataset for analysis after convergence diagnostics confirmed stability. The missing data rate of baseline variables is presented in Figure S1.

Comment: 7. Cutoff selection and clinical thresholds (Youden index) - risk of overfitting and lack of validation.

Response: We appreciate the reviewer’s valuable comment. After applying a stricter screening for PaO₂ measurements as suggested, the shape of the RCS curve showed a slight change. In this study, we did not use the Youden index to determine cutoffs. Instead, we performed restricted cubic spline (RCS) analyses to explore the nonlinear association between the PaO₂/RDW ratio and 7-day mortality. The RCS curve revealed a U-shaped relationship, and the inflection point (lowest estimated hazard) was selected as the reference point for subsequent analyses. Similarly, in the analysis of PaO₂/RDW and invasive mechanical ventilation, the lowest point on the spline curve was used as the reference value. Additionally, we divided the PaO₂/RDW ratio into tertiles to verify the trend across groups. The results showed that the highest and middle tertiles had no statistically significant difference, whereas the lowest tertile was associated with the highest risk of adverse outcomes. Therefore, our findings were based on data-driven distributional grouping and spline-based reference points, rather than on cutoff values derived from the Youden index.

Comment: 8. Modelling of mechanical ventilation as a binary logistic outcome may be inappropriate.

Response: Replaced logistic regression with cause-specific Cox model using death as competing event (Table 3, Figure 6).

Comment: 9. Feature selection and circularity concerns.

Response: We appreciate the reviewer’s comment. To address potential collinearity among predictors, we generated a correlation heatmap and conducted variance inflation factor (VIF) iterations to identify highly correlated variables (Figure 2, Table S1). Variables with VIF values greater than 5 or correlation coefficients exceeding 0.9 were carefully reviewed and removed when appropriate, while clinically important variables were retained.

Comment: 10. External validity, calibration, and clinical implementation not demonstrated.

Response: We appreciate the reviewer’s constructive comment. We fully acknowledge the importance of discrimination, calibration, and external validation in predictive research. However, this study aimed primarily to investigate the association between the PaO₂/RDW ratio and short-term outcomes rather than to develop or validate a predictive model. Therefore, formal discrimination and calibration analyses (such as C-index, AUC, or calibration plots) were not performed. Nevertheless, to ensure robustness, we used multiple complementary regression models (Cox, cause-specific Cox) and confirmed that the associations remained consistent across sensitivity analyses. We have added a statement in the Discussion section to clarify this point and acknowledge that future multicenter prospective studies are warranted to evaluate the predictive performance, calibration, and generalizability of the PaO₂/RDW ratio in different clinical settings.

### Minor Comments

Comment: Terminology and consistency: Use PaO₂ or PO₂ consistently (PaO₂ is standard). Define RDW units (RDW-CV vs RDW-SD). Clarify if PO₂ is arterial (ABG) PaO₂ not SpO₂.

Response: We appreciate the reviewer’s careful observation. We have revised the terminology throughout the manuscript to ensure consistent use of PaO₂, which refers to arterial oxygen partial pressure obtained from arterial blood gas (ABG) measurements in the MIMIC-IV database. In addition, we have clarified that RDW in this study corresponds to RDW-CV (%), as reported in the MIMIC-IV laboratory records, rather than RDW-SD (fL). These definitions and units have been explicitly stated in the revised Methods section for clarity and consistency.

Comment: Statistical reporting: Use consistent formatting for p-values and CIs (e.g., p = 0.017, HR 0.86 [95% CI 0.78–0.95]). Avoid phrases like “P-overall” without explanation - define tests used.

Response: We thank the reviewer for this helpful suggestion. We have carefully reviewed and standardized all statistical reporting throughout the manuscript. P-values are now consistently reported in the format p = 0.XXX, and confidence intervals are presented as HR (95% CI, lower–upper). In addition, we have clarified the meaning of “P-overall” in the figure legends and Methods section — it refers to the overall p-value for trend or group comparison derived from the corresponding Cox proportional hazards or logistic regression models. These revisions ensure consistent and transparent statistical reporting in accordance with journal guidelines.

Comment: Subgroup analyses: State that subgroup analyses are exploratory, adjust for multiplicity (or state they are unadjusted), and present N in each subgroup. The stroke interaction (p = 0.028) needs cautious language and possible biological hypothesis.

Response: The updated subgroup analyses did not reveal any abnormal or inconsistent results. The sample size (N) for each subgroup has been clearly displayed in the corresponding figure.Comment: Ethics and R packages.

Comment: Ethics / data access: You appropriately state MIMIC-IV access; also add a statement on data/code availability and R packages and versions used (you cite R 4.4.1 - also list packages e.g., survival, rms, Boruta, SHAP).

Response: We thank the reviewer for this constructive suggestion. In accordance with the comment, we have added a detailed statement on data and code availability, as well as the software environment used for analysis. Specifically, all analyses were conducted using R software (version 4.4.1; R Foundation for Statistical Computing, Vienna, Austria). The main packages included survival (v3.6-4), rms (v6.8-0), Boruta (v8.0.0), randomForest (v4.7-1.2), mice (v3.16.0), ggplot2 (v3.5.1), SHAPforxgboost (v0.1.3), and forestplot (v3.1.1). A corresponding description has been added to the Availability of data and materials section, indicating that the MIMIC-IV dataset is publicly available on PhysioNet (DOI: 10.13026/kpb9-mt58) and that analytic codes are available from the corresponding author upon reasonable request.

Comment: Limitations: Add explicit limitations section (single-center design proxy via MIMIC, residual confounding, measurement timing, no causal inference, need for external validation, changes in ARDS care across 2008–2022).

Response:
We thank the reviewer for this valuable comment. In accordance with the suggestion, we have added a dedicated Limitations section to the revised manuscript. The section now explicitly acknowledges that:
(1) MIMIC-IV represents a single-center database derived from a tertiary academic medical center;
(2) residual confounding may remain despite extensive covariate adjustment;
(3) the timing of PaO₂ and RDW measurements may vary across patients;
(4) causal relationships cannot be inferred from this retrospective observational design;
(5) external validation using multicenter datasets is warranted; and
(6) changes in ARDS definitions and management practices between 2008 and 2022 may influence the generalizability of our findings.

Comment：Causal claims: Avoid language implying that the ratio “guides” ventilator use or “improves outcomes” without prospective interventional evidence. Rephrase to “may help risk-stratify and identify patients who warrant closer monitoring; prospective validation is required before clinical implementation.”

Response: We thank the reviewer for the helpful suggestion. To avoid overstating causality, we have revised the manuscript to clarify that this study demonstrates associations rather than causal effects. Terms implying causation (e.g., “predictor,” “effect,” or “impact”) were replaced with neutral wording such as “association” or “relationship” throughout the text.

## Conclusion

We sincerely thank the Academic Editor and both reviewers for their thorough evaluation and constructive feedback. We believe the revised manuscript fully addresses all concerns and now meets PLOS ONE’s publication criteria.
