# Supplementary material for: Association of the PaO2/RDW ratio with 7-day mortality and risk of early invasive mechanical ventilation in ICU patients with delirium associated with ARDS: A retrospective cohort study from the MIMIC-IV database
Source: PLoS One. 2025 Dec 19;20(12):e0339390. doi: 10.1371/journal.pone.0339390 (PMC12716735; doi:10.1371/journal.pone.0339390)
Supplement: Attachment — Submitted filename: Response_to_Reviewers_auresp_2.docx [file pone.0339390.s005.docx]

**Response to the Academic Editor**

Manuscript ID: PONE-D-25-24350R1

Title: Association of the PaO₂/RDW Ratio With 7-Day Mortality and Risk of Early Invasive Mechanical Ventilation in ICU Patients With Delirium Associated With ARDS: A Retrospective Cohort Study From the MIMIC-IV Database

## Comment from the Academic Editor

Take a look to the order of the authors. In the revised manuscript is differed from that in the first submission. It requires correction.

## Response

We sincerely thank the Academic Editor for pointing out this issue. The difference in author order between the previous and original submissions was due to an unintentional clerical error during the last revision.

The correct author sequence is:
Jiao Xu², Jun Jin¹, Shan Zou¹, Si-hao Zheng¹, Qing-shan Zhou¹, and Jiang-Tao Deng¹*

All authors have reviewed and approved this correction, and their contributions and authorship designations remain unchanged. We sincerely apologize for the oversight and have corrected the author list accordingly in the revised manuscript.

Sincerely,
Jiang-Tao Deng, MD, PhD
(On behalf of all co-authors)
Department of Critical Care Medicine
The University of Hong Kong–Shenzhen Hospital
Email: lopdeng005@gmail.com
